# Acupuncture Improves White Matter Perfusion and Integrity in Rat Model of Vascular Dementia: An MRI-Based Imaging Study

**DOI:** 10.3389/fnagi.2020.582904

**Published:** 2020-11-23

**Authors:** Si-Ming Ma, Lu Wang, Xin-Tong Su, Na-Na Yang, Jin Huang, Lu-Lu Lin, Jia-Kai Shao, Jing-Wen Yang, Cun-Zhi Liu

**Affiliations:** ^1^Beijing Hospital of Traditional Chinese Medicine Affiliated to Capital Medical University, Beijing, China; ^2^International Acupuncture and Moxibustion Innovation Institute, School of Acupuncture-Moxibustion and Tuina, Beijing University of Chinese Medicine, Beijing, China

**Keywords:** acupuncture, ASL, DTI, cerebral blood flow, white matter, microglial activation

## Abstract

White matter lesions induced by chronic cerebral hypoperfusion are associated with cognitive impairment in vascular dementia (VaD). Previous studies have shown that acupuncture can ameliorate the cognitive deficits of individuals with VaD. However, the neuroimaging mechanisms of acupuncture on white matter perfusion and integrity remain elusive. In this study, the VaD model was induced by bilateral common carotid arteries occlusion (BCCAO) in rats. Novel object recognition task and Morris water maze were performed to evaluate short-term memory and spatial learning and memory. Arterial spin labeling and diffusion tensor imaging (DTI) were used to measure the cerebral blood flow (CBF) and the white matter integrity. Pathological examinations detected the myelin loss and concomitant neuroinflammation. The results demonstrate that BCCAO rats with reduced CBF exhibited worse performance and altered DTI parameters, including decreased fractional anisotropy, increased radial diffusivity, and axial diffusivity in white matter regions. Acupuncture ameliorated cognitive impairment, increased CBF, and protected the myelin sheath integrity but not the axons of BCCAO rats. These protective effects of acupuncture on white matter were significantly correlated with improved CBF. Pathological examination confirmed that the loss of myelin basic protein and microglial accumulation associated IL-1β and IL-6 production were attenuated by acupuncture treatment. Our findings suggest that acupuncture protects cognitive function of BCCAO rats by improving white matter perfusion and integrity.

## Introduction

Vascular dementia (VaD) is the second most common cause of dementia after Alzheimer’s disease (Gorelick et al., 2011). VaD is characterized by hemodynamic alterations, including persistent cerebral blood flow (CBF) attenuation and dysfunction of CBF regulation (Giezendanner et al., 2016; Ogoh, 2017). The circulatory abnormalities are mainly attributed to the aging vasculature failing to cope with biological demands, which results in subcortical ischemia and white matter lesions (Alber et al., 2019). Ample evidence suggests that chronic cerebral hypoperfusion (CCH) is a leading pathophysiological mechanism of white matter lesions in VaD (Iadecola, 2013; Dichgans and Leys, 2017; Alber et al., 2019). Cerebral hypoxia-ischemia induced by CCH is sufficient to trigger white matter inflammation and oxidative stress (Dong et al., 2011; Miyamoto et al., 2013), which, in turn, impact the regulation of the cerebral blood supply (Faraci, 2011; Iadecola, 2013). Such an imbalance of CBF regulation would aggravate white matter injury and contribute to the loss of working memory or executive dysfunction (Calabrese et al., 2016; Du et al., 2017).

White matter is a subcortical structure that consists of neuronal axons, the myelin sheath, and the surrounding glial cells. Due to the deep location at non-overlapping regions of cerebral arterioles or capillaries, white matter is vulnerable to the CBF reductions (Wang et al., 2020). White matter lesions caused by ischemia, encompassing axonal damage, loss of myelin-associated glycoproteins, and microglial activation, are common pathological changes in VaD (Struys et al., 2017; Qin et al., 2018; Zhang et al., 2020). Diffusion tensor imaging (DTI) study reports that VaD patients have a specific pattern of white matter lesions affecting the thalamic radiations or corpus callosum (CC) to a greater extent (Fu et al., 2012; Palesi et al., 2018), which appears as decreased fractional anisotropy (FA), increased radial diffusivity (RD), and axial diffusivity (AD) (Choi et al., 2016; Ben-Ari et al., 2019). Further perfusion imaging studies demonstrate that VaD patients develop white matter lesions associated with persistent reduction of parenchymal CBF (Schuff et al., 2009), which remains at 50% of the baseline level for at least 2 weeks (Hattori et al., 2016).

Acupuncture is a non-pharmacologic therapy of traditional Chinese medicine that originated from ancient clinical practice. It is performed at stimulation points or meridians spread over the surface of body and within many organs (Adams et al., 2011). Clinical studies confirm that VaD patients treated with acupuncture exhibit improvement of cognitive status (memory, orientation, calculation, and self-managing ability), and the restored cortical CBF might underlie the therapeutic effects (Yu et al., 2006; Shi et al., 2014; Uchida and Kagitani, 2015). It has been shown that the beneficial effect of acupuncture on CBF is associated with promotion of the cholinergic vasodilative system or suppression of oxidative stress in the cortex of rats (Zhang et al., 2014; Uchida and Kagitani, 2015). Although this research investigated the role of acupuncture in cortical blood flow and cognitive performance microscopically, the role of neuroimaging mechanism of acupuncture in white matter perfusion and structural integrity has not been delineated.

In this study, we used arterial spin labeling (ASL) to monitor CBF changes in the CC and external capsule (EC) in rats with bilateral common carotid artery occlusion (BCCAO) and acupuncture treatment. White matter lesions in the CC, EC, internal capsule (IC), optic tract (OT), and optic nerves (ON) were detected by DTI to evaluate the disruption of white matter integrity. Additionally, pathological changes in white matter and expression levels of neuroinflammatory markers were also analyzed to validate the features of myelin loss and inflammatory responses.

## Materials and Methods

### Animals

**FIGURE 1:**
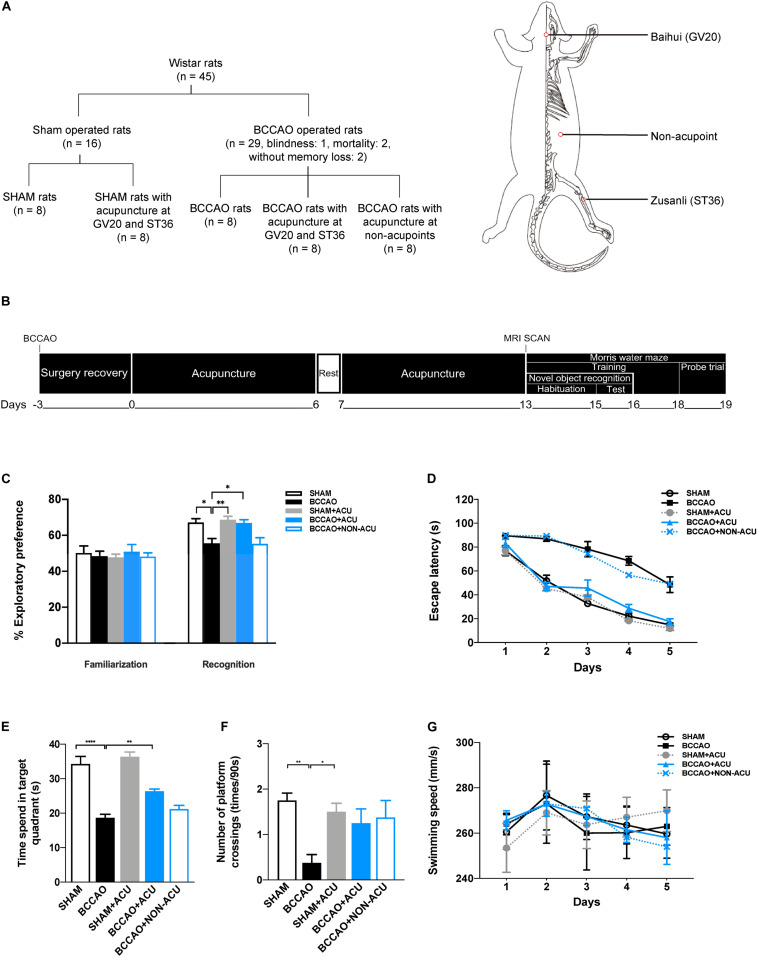
Acupuncture Ameliorates Short-term and Spatial and Learning Memory of BCCAO-Induced CCH Rats. **(A)** Animal groups and the location of acupoints. Red circles indicate the location of Baihui (GV20), Zusanli (ST36), and non-acupoints. **(B)** Experimental procedures. **(C)** Percentage of exploratory preference of rats in familiarization and recognition test. **(D)** Escape latency. **(E)** Time spent in the target quadrant. **(F)** Number of platform crossings. **(G)** Swimming speed. **(D,E,F,G)** were measured in SHAM, BCCAO, SHAM + ACU, BCCAO + ACU, and BCCAO + NON-ACU groups using novel object recognition task and Morris water maze. Data are presented as mean ± SEM (n = 8 per group). ^∗^*p* < 0.05, ^∗∗^*p* < 0.01, ^****^p < 0.0001, respectively. SHAM, SHAM-operated group; BCCAO, BCCAO-operated group; BCCAO + ACU, BCCAO-operated + acupuncture at GV20 and ST36 group; BCCAO + NON-ACU, BCCAO-operated + acupuncture at non-acupoints group.

Wistar rats (*n* = 45, male, 260–280 g) were housed on a 12/12 h light/dark cycle with controlled temperature (24 ± 0.5°C) in the animal facility at Dongfang Hospital, Beijing University of Chinese Medicine. Food and water were available *ad libitum* for the rats. The rats were randomized into five groups by a digital table (Figure 1A): sham-operated rats (SHAM, *n* = 8), rats subjected to BCCAO (BCCAO, *n* = 8), SHAM rats treated with acupuncture (SHAM + ACU, *n* = 8), BCCAO rats treated with acupuncture (BCCAO + ACU, *n* = 8), and BCCAO rats treated with non-acupoint acupuncture (BCCAO + NON-ACU, *n* = 8). Surgery caused one blindness, and the mortality rate was 4%. The rat blinded due to surgery, as well as two BCCAO-operated rats without impairment of working memory that was validated by behavioral tests, were not included in the experiment. All experiments complied with the procedures of the US National Institutes of Health Guide for the Care and Use of Laboratory Animals, and were approved by the Institutional Animal Care and Use Committee of Dongfang Hospital, Beijing University of Chinese Medicine (ethics approval number 201822).

### Surgical Procedures

The VaD model of rat was induced by the BCCAO method as preciously described (Ye et al., 2017). In detail, rats were anesthetized with an intraperitoneal injection of sodium pentobarbital (40 mg/kg bodyweight). A ventral cervical midline incision was made, and both common carotid arteries were exposed and permanently double ligated with 4-0 silk sutures. In the SHAM group, the same operation was performed as in the BCCAO group except for artery occlusion. Before and the 2 consecutive days after the operation, 2% lidocaine (0.1 ml/day) was injected intramuscularly to relieve the postoperative pain.

### Acupuncture Treatment

Beginning 3 days after surgery, the awake rats received 2 weeks of acupuncture therapy, once daily, and one day of rest after six treatments. Acupuncture needles (Hwato, China, 0.22 × 5 mm) were used for stimulation. In the BCCAO + ACU group, treatment was performed at “Baihui” (GV20) (located at the midline of the head, approximately midway on the line connecting the apices of the auricles) and bilateral “Zusanli” (ST36) (located at 2 mm lateral to the anterior tubercle of the tibia and 5 mm below the capitulum fibulae under the knee joint) acupoints synchronously (Figure 1A). The selection of acupoints was based on our previous study in VaD rats, which suggests that the combination of GV20 + ST36 has better attenuating effects on cognitive dysfunction (Ye et al., 2017). For the BCCAO + NON-ACU group, bilateral non-acupoints (on the bilateral hypochondrium, 10 mm above the iliac crest) were chosen as the insertion site (Figure 1A). The needles were retained for 15 min before removal. The same catching-grasping stimulation used for the BCCAO + ACU group was used for all the other non-treatment groups.

### Novel Object Recognition Task (NORT)

The short-term recognition memory was assessed using the NORT (Figure 1B) according to previously published protocol (Bevins and Besheer, 2006). The animals were habituated to an opaque testing arena (40 cm × 40 cm × 40 cm) on 2 consecutive days, for 30 min each time. On day 3, the rats were individually placed in the arena and allowed to explore two objects for 5 min before being returned to their home cages (familiarization). After 1 h, one object was replaced by a new one of similar size and distinct color. Rats were placed in the arena and allowed to explore the objects again for 2 min (recognition). Once the center of the rat head was oriented within 45° and 4 cm of the novel object, it would be registered as one novel object exploration. Leaning against, turning around, or sitting on an object was regarded as an invalid exploration. A video camera was fixed above the arena to record the trials. The objects and arena were cleaned with ethanol following each test to avoid the influence of olfactory trails. The percentage exploratory preference was calculated as time exploring novel object/(time exploring old object + time exploring novel object) × 100%.

### Morris Water Maze Test

The Morris Water Maze (MWM) consists of a circular pool 160 cm in diameter. It was divided into four quadrants. During the training, all rats were subjected to three training trials per day for 5 consecutive days. In each trial, animals were allowed 90 s to search for the submerged platform and 10 s to remain on it. The latency to escape and swimming speed were recorded. On day 6, the platform was removed to conduct a probe trial, and the rats were permitted to swim freely for 90 s. The time spent in the target quadrant and the number of platform crossings were recorded. The experimental procedure is shown in Figure 1B.

### MRI Acquisition

MRI scanning was arranged on the 13th day after BCCAO surgery. Rats were induced with anesthesia with 2% isoflurane and maintained on a mixture of 2% isoflurane and 98% oxygen during scanning. All images were acquired with a 7.0 T MR scanner (Bruker BioSpin GmbH, Rheinstetten, Germany). Coronal T_2_WI rapid acquisition with relaxation enhancement (RARE) was performed with the following parameters: repetition time (TR) = 5000 ms; echo time (TE) = 36 ms; flip angle = 180°; slice thickness = 0.7 mm; field-of-view (FOV) = 3.5 × 3.5 cm; matrix = 256 × 256. Perfusion images were obtained using continuous ASL with echo-planar imaging fluid-attenuated inversion recovery (EPI-FLAIR) sequence with the following parameters: TR = 18000 ms; TE = 25 ms; flip angle = 90°; slice thickness = 2 mm; FOV = 3.0 × 3.0cm; matrix = 128 × 128. Acquisition of DTI using spin echo planner imaging (SE) sequence was set by TR = 6250 ms; TE = 22 ms; flip angle = 90°; slice thickness = 0.8 mm; FOV = 3.0 × 3.0 cm; matrix = 128 × 128; b values = 1000 s/mm^2^ along with a total of 30 diffusion gradient directions.

### Data Processing

#### CBF and DTI Parameters Calculation

Arterial spin labeling images were reconstructed automatically with paravision v5.1 software (Bruker Biospin, Germany). The CC and EC were drawn manually according to T2WI images to measure the CBF.

**FIGURE 2:**
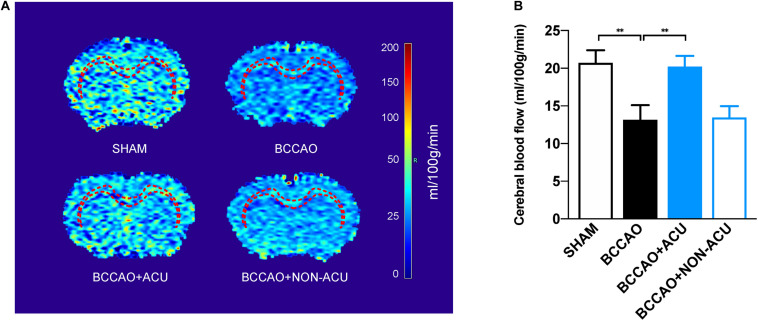
Acupuncture Improves White Matter Perfusion of BCCAO-Induced CCH Rats. **(A)** Reconstructed cerebral blood flow map and the white matter regions of interest (corpus callosum and external capsule, red dotted line). **(B)** The quantification of cerebral blood flow of white matter regions by arterial spin labeling. Data are presented as mean ± SEM (*n* = 8 per group). ^∗∗^*p* < 0.01. SHAM, sham-operated group; BCCAO, BCCAO-operated group; BCCAO + ACU, BCCAO-operated + acupuncture at GV20 and ST36 group; BCCAO + NON-ACU, BCCAO-operated + acupuncture at non-acupoints group.

Diffusion tensor imaging raw data were processed using DSI Studio Software^1^. Files of 2D sequence were loaded directly into the software for distortion correction. The gradient magnetic field (*b*-value) and gradient magnetic field vector (*b*-vector) were added for flip of x-, y-, or z-coordinates and eddy correction. For the reconstruction, a brain mask was generated using a DTI method to filter out the background region (Basser et al., 1994). The CC, EC, internal capsule (IC), optic tract (OT), and optic nerves (ON) were selected as regions of interest (ROIs), drawn manually according to the Paxinos rat brain atlas (Figure 2A; Watson and Paxinos, 1996). DTI parameters, including fractional anisotropy (FA), axial diffusivity (AD), radial diffusivity (RD), and mean diffusivity (MD), were calculated. All imaging data were processed by one independent analyst.

#### White Matter Fiber Tracking

Fiber tracking was conducted to depict the fiber numbers and length in four groups. Fiber tracts were seeded from CC and EC in each rat. The tracking parameters was set as follows: tracking index: FA; angular threshold: 45°; step size: 0.05 mm; smoothing: 0.01; min length: 0.1 mm; max length: 30 mm; seed orientation: primary. The fiber density (fiber number/voxel number) and fiber length were calculated according to the previous study (Choi et al., 2016).

### Immunohistochemistry

After the behavioral testing, all rats were injected with an overdose of sodium pentobarbital (100 mg/kg) and decapitated. The brain tissues of the rats were collected and embedded in an optimal cutting temperature compound. The frozen brain tissue was sectioned with a freezing microtome to a thickness of 10 μm. Brain sections were washed in PBS, blocked with 3% hydroxide, and incubated with primary antibodies to myelin basic protein (MBP, 1:100, Abcam), ionized calcium binding adapter molecule1 (Iba-1, 1:1000, Abcam), and glial fibrillary acidic protein (GFAP, 1:1000, Abcam) at 4°C overnight. Subsequently, the sections were incubated with biotinylated secondary antibodies (1:100, ZSGB-BIO) and 3,3′ diaminobenzadine tetrahydrochloride (DAB) as a chromogenic substrate (Solarbio). DAB immunolabeled sections were captured using an optical microscope (Nikon DS-Ri2). All measurements were analyzed blindly by a researcher using ImageJ v2.0.0 software.

### Cytokine Analyses

For the analyses of neuroinflammatory markers, the levels of tumor necrosis factor-α (TNF-α), interleukin-1β (IL-1β), and interleukin-6 (IL-6) in white matter were quantified using ELISA kit (TNF-αand IL-1β: Proteintech, CN; IL-6: Keygentech, CN) according to the manufacturer’s instructions. In detail, white matter tissue was homogenized and centrifuged at 10000 rpm for 15 min at 4°C. A total of 100-μl sample was added into each well and incubated at 37°C for 120 min. After washing, the plate was incubated in 100 μl detection antibody at 37°C for 60 min and in 100 μl HRP-conjugate antibody at 37°C for 40 min, respectively. Then 50 μl of color agent at 37°C was added to the plate for 20 min and then treated with stop buffer to stop the reaction. The optical density values of the samples at 450 nm were detected to calculate the concentration. All samples were analyzed in duplicate.

### Western Blot

White matter tissues were lysed with cold radio immunoprecipitation assay lysis buffer solution (Beyotime, CN) supplemented with a phosphatase inhibitor (Thermo, United States) and protease inhibitor (Thermo, United States). Proteins (40 μg) were loaded onto a 15% SDS-PAGE gel for electrophoresis and transblotted to polyvinylidene difluoride membrane (Merck Millipore, United States). Then the membranes were blocked with 5% non-fat milk in solution of tris buffered saline with Tween and incubated overnight at 4°C with primary antibodies MBP (rabbit anti-MBP, 1:1000, ProteinTech, CN) and β-actin (rabbit anti-β-actin, 1:1000, Biodragon, CN). Then the membranes were incubated in the dark with Delight 800 conjugate anti-rabbit (H + L) second antibody (1:5000, KPL, United States). The images were scanned with an imaging system (Bio-Rad, United States) and analyzed using Image J software.

### Statistical Analysis

Statistical analysis was performed using SPSS v22.0 software. Graphs were generated by Graphpad Prism 8 software. Results of the MWM testing were analyzed using two-way repeated measures ANOVA. The NORT, CBF, DTI parameters, pathological indices, cytokine, and protein expression were compared between groups using one-way ANOVA followed by *post hoc* Tukey’s test. Correlation analysis between CBF and DTI parameters, including FA, RD, and fiber length, was conducted with a two-tailed Pearson’s correlation. All data are presented as mean ± standard error of mean (SEM), and *p* < 0.05 was set statistically significant.

## Results

### Effects of Acupuncture on Cognitive Impairments in BCCAO-induced CCH Rats

We initially assessed the short-term memory of rats using the NORT (Figure 1C). In the familiarization test, no significant preference was found between groups when the rats explored two novel objects (SHAM: 67 ± 2%, BCCAO: 56 ± 3%, SHAM + ACU: 69 ± 2%, BCCAO + ACU: 67 ± 2%, BCCAO + NON-ACU: 55 ± 3%, p = 0.942). On the contrary, 56 ± 3% preference for new location of object in BCCAO rats, compared with 67 ± 2% preference in the SHAM group (*p* = 0.017), indicates failing memorization. After acupuncture treatment, the preference for the novel object was increased to 67 ± 2% compared with BCCAO rats (*p* = 0.021), suggesting that the short-term memory is restored. However, acupuncture at non-acupoints was not sufficient to increase the time of exploring novel subject for BCCAO rats (*p* > 0.05). In addition, there was no effect of acupuncture treatment on behavioral performance of sham-operated rats (*p* > 0.05).

Then we performed the MWM to examine spatial learning and memory (Figures 1D–G). A significant time effect (*F* [4, 140] = 184.6, *p* < 0.0001), treatment effect (*F* [4, 35] = 80.1, *p* < 0.0001), and interaction of time and treatment (*F* [16, 140] = 4.473, *p* < 0.0001) were found in the escape latency (Figure 1D). In probe trial, BCCAO rats (48.46 ± 16.12 s) spent more time finding the hidden platform than the SHAM group (14.85 ± 1.41 s, *p* < 0.0001). The rats treated with acupuncture at ST36 and GV20 showed a better escape latency (17.56 ± 2.27 s) than BCCAO rats (*p* < 0.0001). For the time spent in the target quadrant (Figure 1E), there was a marked decrease in the BCCAO group (18.64 ± 0.96 s) compared with the SHAM group (34.29 ± 2.03s, *p* < 0.0001). With acupuncture at GV20 and ST36, a significant increase in the time spent in the target quadrant (26.34 ± 00.64 s, compared with the BCCAO group, *p* = 0.007) was observed. The number of platform crossings in the BCCAO group (0.38 ± 0.17 times/90 s) was decreased compared with the SHAM group (1.75 ± 0.15 times/90 s, *p* = 0.005), but acupuncture did not show improvement in the decreased crossing number (1.25 ± 0.29 times/90 s, *p* > 0.05, Figure 1F). No significant difference was observed between the BCCAO + NON-ACU group (escape latency: 49.1 ± 6.06 s, time spend in target quadrant: 21.16 ± 1.01 s, number of platform crossings: 1.38 ± 0.35 times/90 s) and the BCCAO group in escape latency (*p* > 0.05), time spend in target quadrant (*p* > 0.05), and the number of platform crossings (*p* = 0.069). In addition, among the five groups, there was no significant time effect (*F*[4, 140] = 1.198, *p* = 0.315), treatment effect (*F*[4, 35] = 0.019, *p* > 0.05), or interaction of time and treatment (*F*[16, 140], *p* > 0.05) in swimming speed (Figure 1G), suggesting no effect on motor function.

### Effects of Acupuncture on the White Matter CBF

**FIGURE 3:**
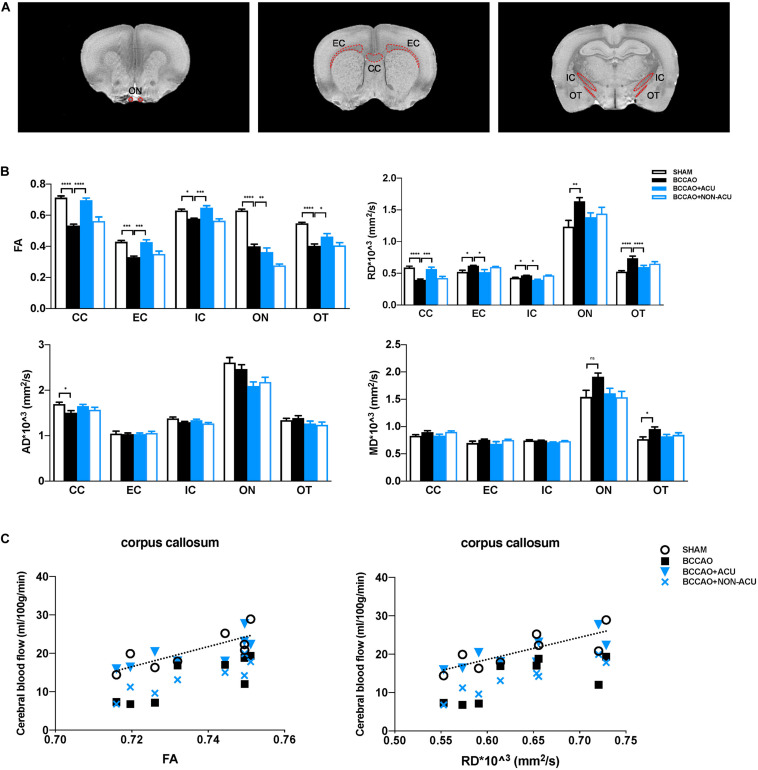
Acupuncture Protects White Matter Integrity from Cerebral Chronic Hypoperfusion of BCCAO Rats. **(A)** The white matter ROIs drawn from T2WI images of the rat brain (red dotted line). **(B)** Quantification of DTI parameters in the white matter ROIs in SHAM, BCCAO, BCCAO + ACU, and BCCAO + NON-ACU groups using diffusion tensor imaging. **(C)** In the corpus callosum, the two-tail Pearson’s correlation analysis revealed significant positive correlation between CBF and FA and partial negative correlation between CBF and RD. Values are presented as mean ± SEM (*n* = 8 per group). ^∗^*p* < 0.05, ^∗∗^*p* < 0.01, ^∗∗∗^*p* < 0.001, ^****^p < 0.0001, respectively. FA, fractional anisotropy; RD, radial diffusivity; AD, axial diffusivity; MD, mean diffusivity; CC, corpus callosum; EC, external capsule; IC, internal capsule; ON, optic nerve; OT, optic tract; SHAM, sham-operated group; BCCAO, BCCAO-operated group; BCCAO + ACU, BCCAO-operated + acupuncture at GV20 and ST36 group; BCCAO + NON-ACU, BCCAO-operated + acupuncture at non-acupoints group.

The location of ROIs, including CC and EC, was marked in brain heatmaps (Figure 3A). As shown in Figure 3B, CBF in CC and EC was significantly decreased to 13.16 ± 1.81 ml/100 g/min in BCCAO rats (*p* = 0.015, compared with 20.71 ± 1.57 ml/100 g/min in the SHAM group). However, in the BCCAO + ACU group, white matter CBF improved to 20.21 ± 1.33 ml/100 g/min (*p* = 0.026, compared with the BCCAO group). There was no significant difference between the BCCAO and BCCAO + NON-ACU groups (*p* > 0.05, CBF of BCCAO + NON-ACU: 13.45 ± 1.41 ml/100 g/min), which means that non-acupoint acupuncture did not protect the white matter from CCH.

### Effects of Acupuncture on White Matter Integrity in DTI

**FIGURE 4:**
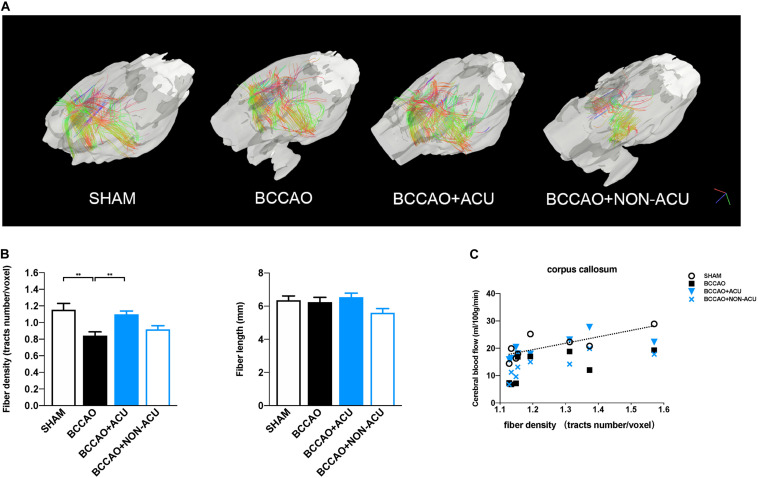
Visualization and Quantification of White Matter Tracts Indices in Rats. **(A)** Visualization of corpus collosum–associated white matter regions by reconstructing DTI images. **(B)** quantification of whiter matter fiber density (fiber number/voxel) and fiber length in SHAM, BCCAO, BCCAO + ACU, and BCCAO + NON-ACU groups. Data are presented as mean ± SEM (*n* = 8 per group). **(C)** The two-tail Pearson’s correlation coefficients between CBF and fiber density in the corpus callosum. ^∗∗^*p* < 0.01. SHAM, sham-operated group; BCCAO, BCCAO-operated group; BCCAO + ACU, BCCAO-operated + acupuncture at GV20 and ST36 group; BCCAO + NON-ACU, BCCAO-operated + acupuncture at non-acupoints group.

Fractional anisotropy, RD, AD, and MD values in CC, EC, IC, ON, and OT were calculated using DSI studio (Figure 2A). As shown in Figure 2B and Supplementary Table 1, a significant decrease in FA and increase in RD in all representative ROIs were observed in the BCCAO rats in comparison to the SHAM group (CC: *p* < 0.0001, EC: *p* < 0.0001, IC: *p* = 0.011, ON: *p* < 0.0001, OT: *p* < 0.0001), suggesting extensive damage of white matter integrity. After 2 weeks of acupuncture treatment, the changes in FA and RD induced by BCCAO was reversed (except the RD value in ON, CC: *p* < 0.0001, EC: *p* = 0.0002, IC: *p* = 0.0004, ON: *p* = 0.002, OT: *p* = 0.049). In the BCCAO + NON-ACU group, no improvement was observed in FA and RD of all ROIs. Statistical differences of AD in CC (*p* = 0.039) and MD in OT (*p* = 0.012) were also found between SHAM and BCCAO groups. But these two DTI indices were not affected by acupuncture (AD in CC: *p* = 0.156, MD in OT: *p* = 0.101). Results of fiber tracking demonstrate that a prominent reduction in fiber density induced by BCCAO (*p* = 0.002) was attenuated by acupuncture (*p* = 0.011), while the fiber length was not affected by surgery or acupuncture treatment (*p* = 0.086, Figures 4A,B). In addition, Pearson’s correlation analysis (the data are listed in Supplementary Table 2) reveals that CBF was positive correlated with FA values (Figure 2C, *r* = 0.793, *r* = 0.818, *r* = 0.765, *r* = 0.898, respectively), partially correlated with white matter fiber density (Figure 4C, *r* = 0.778, *r* = 0.607, *r* = 0.697, *r* = 0.776, respectively), and partially negative correlated with RD values (Figure 2C, *r* = 0.79, *r* = 0.689, *r* = 0.81, *r* = 0.957, respectively) in four groups.

### Effects of Acupuncture on White Matter Pathology

**FIGURE 5:**
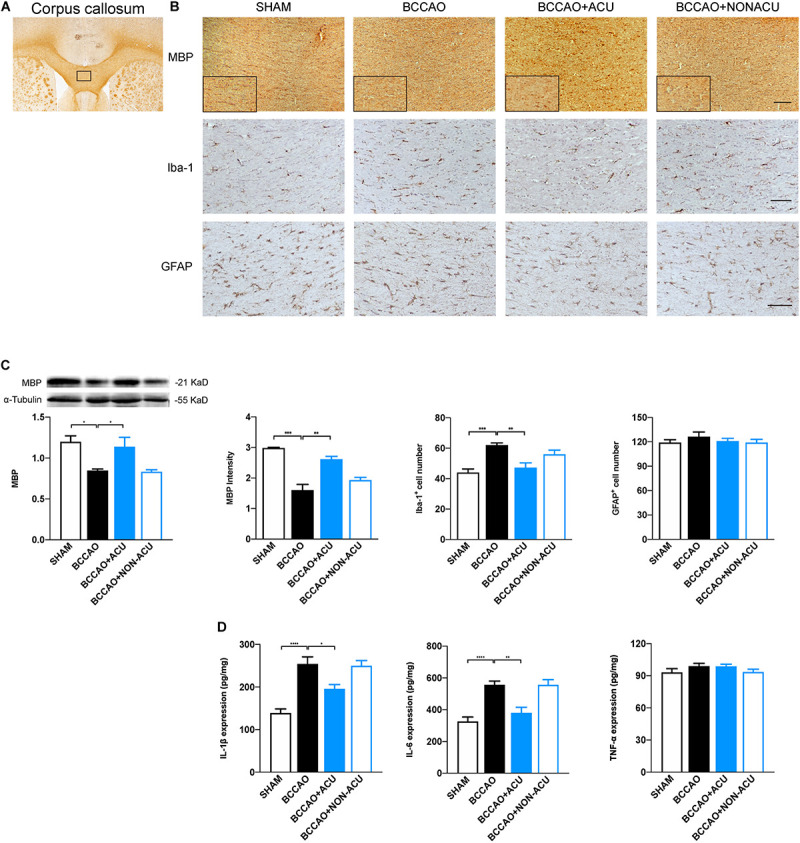
Acupuncture Attenuates Loss of Myelin Sheath and Microglia Activation in BCCAO-Induced CCH Rats. **(A)** ROI of corpus callosum for immunohistochemistry (black rectangular box). **(B)** Representative and magnification (black rectangular boxes) immunostaining of MBP, Iba-1^+^, and GFAP^+^ glia in four groups. **(C)** Quantification of protein level of MBP, calculations of MBP^+^ area, Iba-1^+^ cell number, and GFAP^+^ cell number. **(D)** Quantification of neuroinflammatory markers including IL-1β, IL-6, and TNF-α in white matter of SHAM, BCCAO, BCCAO + ACU and BCCAO + NON-ACU groups. Scale bar: 50 μm, data are presented as mean ± SEM (*n* = 6 per group). ^∗^*p* < 0.05, ^∗∗^*p* < 0.01, ^∗∗∗^*p* < 0.001, ^****^p < 0.0001. MBP, myelin basic protein; Iba-1, ionized calcium binding adapter molecule 1; GFAP, glial fibrillary acidic protein; SHAM, SHAM-operated group; BCCAO, BCCAO-operated group; BCCAO + ACU, BCCAO-operated + acupuncture at GV20 and ST36 group; BCCAO + NON-ACU, BCCAO-operated + acupuncture at non-acupoints group.

To evaluate the pathological changes of all types of glia in the white matter region, we quantified the expression of myelin basic protein (MBP, located at membrane of oligodendrocyte), Iba-1, and GFAP (Figures 5A–C). We found that the positive expression area and the quantification of MBP were reduced significantly at the 14th day after BCCAO surgery (compared with the SHAM group, positive area: *p* = 0.0001, expression: *p* = 0.011), while acupuncture treatment alleviated the MBP loss effectively in CC (compared with the BCCAO group, positive expression area: *p* = 0.001, protein quantification: *p* = 0.039). The significant increased number of Iba-1 positive cells in the CC of the BCCAO group (compared with the SHAM group, *p* = 0.0002) and decreased number of Iba-1 positive cells in the BCCAO + ACU group (compared with BCCAO group, *p* = 0.0007) implicates that prominent neural inflammation in white matter was reduced by acupuncture at GV20 and ST36. However, non-acupoints failed to attenuate microglial accumulation (compared with the BCCAO group, *p* > 0.05). In addition, there was no remarkable variance of GFAP-positive astrocyte number among the four groups (*p* = 0.183).

### Effect of Acupuncture on Neuroinflammation

As neuroinflammation induced by microglia accumulation or activation deteriorates the white matter lesions, we analyzed its contribution to the anti-inflammatory effects of acupuncture (Figure 5D). The quantification of IL-1β, IL-6, and TNF-α in corpus callosum suggests that IL-1β and IL-6 production was elevated in BCCAO (compared with the SHAM group, IL-1β: *p* < 0.0001, IL-6: *p* < 0.0001, TNF-α: *p* = 0.454) and the BCCAO + NON-ACU group (compared with the BCCAO group, IL-1β: *p* > 0.05, IL-6: *p* > 0.05, TNF-α: *p* = 0.483). Acupuncture treatment attenuated the neuroinflammation to the level of the SHAM group (IL-1β: *p* = 0.013, IL-6: *p* = 0.002, TNF-α: *p* > 0.999). The distinct differences in TNF-α level were not found among the four groups (*p* = 0.265).

## Discussion

In this study, our results demonstrate that spatial learning and memory of BCCAO rats could be ameliorated by acupuncture treatment, as evidenced by increased white matter perfusion and integrity. In CC and EC, where the white matter fibers are the most abundant, white matter integrity is correlated with the alteration of CBF. Pathological evidence suggests that the attenuated myelin loss and neuroinflammation partially underlie the beneficial effects of acupuncture on white matter.

Acupoints selection and the course of treatment are key factors that affect the effectiveness of acupuncture. In the current study, GV20 and ST36 were selected, based on our published results, as an ideal combination of acupoints that is beneficial to memory deficits in BCCAO rats (Ye et al., 2017; Xiao et al., 2018). For the course of treatment, outpatients usually take regular rest weekly during the acupuncture. For this reason, a one-day break every 2 weeks is supposed to be consistent with the clinical practice.

Preliminary research provides some clues that the therapeutic effects of acupuncture on visceral function may be mediated by specific peripheral nerves. For example, GV20 is located at the area of trigeminal nerve innervating (Wang et al., 2017), by which the signal of acupuncture stimulation might be transferred to the meninges or brainstem nucleus. For ST36, there is less strong evidence suggesting a direct anatomic connection between the acupoint and brain. But the sciatic nerve is supposed to be a peripheral structure mediating the acupuncture signals from ST36 to the spinal cord, the nucleus tractus solitary and higher center (Torres-Rosas et al., 2014). Although acupuncture at non-acupoints is an invasive stimulation, there is no obvious effect of non-acupoints on animal behavior and white matter pathology in this study. Based on the neuroanatomical evidence, we presume that (1) non-acupoints location are far away from the main trunk of the nerve, and the stimulus signal may not be transduced to the central nerve system successfully (no effect); and (2) the signal derived from acupuncture stimulation at non-acupoint could be transferred to the spinal cord or brain. Nevertheless, they may not be effective acupoints for cognitive dysfunction (non-specific effect).

Large number of studies suggest that spatial learning and memory are impaired in BCCAO rats (Choi et al., 2016; Du et al., 2017; Qin et al., 2018). As a peripheral stimulation, acupuncture could improve the performance of BCCAO rodents in MWM by increasing the CBF velocity and volume of prefrontal lobe and hippocampus (Wang et al., 2015; Ding et al., 2019). Consistent with previous studies, we found that BCCAO rats with increased escape latency and decreased time in target quadrant in probe trials showed reduced white matter CBF. In contrast, rats treated with 2-week acupuncture had better MWM performance and white matter perfusion. These outcomes indicate that acupuncture can improve cognitive dysfunction of VaD rats by increasing the white matter CBF.

Diffusion tensor imaging is a sensitive MRI technique that could highlight the changes in white matter microstructure in terms of diffusion properties of tissue *in vivo* (Palesi et al., 2018). FA and MD represent the white matter organization in general, while RD and AD reflect quality of the myelin sheath and the axon integrity, respectively (Alfaro et al., 2018). Human study indicates that subcortical VaD patients exhibit decreased FA and increased MD in all white matter regions except occipital areas (Kim et al., 2015). But the DTI parameters reported in rats show more heterogeneity, characterized by remarkable alteration of FA, RD, and AD in ON, OT, and EC 2 weeks after BCCAO, or significant reductions in MD, RD, and AD in CC and EC 5 weeks after surgery (Wang et al., 2015; Choi et al., 2016). We combined ASL with DTI and found that predominant reduction in CBF volume is correlated with significant white matter disintegration at 2 weeks after surgery and appears as lower FA and higher RD values in white matter regions, which are in line with previous studies. After acupuncture treatment, FA, RD, and white matter perfusion are significantly ameliorated. Therefore, we conclude that compromised myelin sheath but not neuroaxonal damage could be helped by acupuncture.

Visualizing results explained the specific changes in DTI parameters. In this part, we produced quantitative analysis of white matter structure based on previous study, which suggests the remarkable decreased fiber density and fiber length 4 weeks after BCCAO (Choi et al., 2016). However, only contrasting changes in fiber density, but not fiber length in four groups, were observed, indicating that the wrapped myelin sheath is damaged by ischemia, while neuroaxon structure is still intact. Given that the MRI acquisition was arranged at the 13th day after surgery, we speculate that the axon had not been attacked by hypoperfusion. Taken together, our diffusion imaging outcomes prove in part that acupuncture treatment protects myelin sheath integrity from CCH in rats with subcortical white matter lesions.

It has been suggested that glia-associated neuroinflammatory response aggravates the white matter lesions (Sharma et al., 2010; Datta et al., 2017). In BCCAO rats, both microglia and astrocyte activation contribute to the white matter demyelination (Choi et al., 2016; Saggu et al., 2016). We observed the specific pathological changes in the white matter to evaluate the demyelination and neuroinflammation. In alignment with the DTI results, our findings suggest that the disintegration of white matter, represented by loss of myelin sheath and microglial accumulation-mediated neuroinflammation, could be halted by acupuncture treatment. It is worth noting that astrogliosis was not significant in this study. According to the previous accomplishment, astrocyte activation could lead to the increased neuroinflammatory response and white matter lesions in CCH rats (Saggu et al., 2016). This might be attributed to the different chronic hypoperfusion model, bilateral common carotid artery stenosis, that was used in that experiment. Although we did not observe the astrocyte activation, our study does not rule out the possibility that astrocyte plays a crucial role in the protective effect of acupuncture in white matter lesions. Therefore, further work needs to be done to elucidate the glia-associated neural circuits that acupuncture may regulate in BCCAO-induced CCH rats.

Several limitations need to be considered when explaining our results. First, both behavioral tests and pathological examination were performed in the same cohort of rats. Our data cannot exclude the bias that the stress induced by forcing swimming in MWM may result in unpredictable effects on NORT performance and pathological damage. Second, no functional MRI was conducted in this study. It is widely accepted that subcortical white matter structure underlies the neuronal activity in the cortex (Reijmer et al., 2015). The white matter abnormality may result in the decreased functional connectivity or the rearrangement of local cognitive networks. Previous studies prove that these functional networks could be considered neuroimaging evidence of acupuncture effects on cognitive function (Dhond et al., 2008; Qiu et al., 2010; Cui et al., 2018; Wen et al., 2018). Therefore, future studies aiming at structure and function changes and their interaction will help uncover the mechanism of acupuncture. Third, since this is an imaging study, we focused mainly on the macroscopic changes of brain and did not assume and detect signaling pathways mediating the white matter lesions and microglial activation. In fact, microglial polarization mediated inflammatory and complement pathways contribute to white matter lesions in the VaD model (Qin et al., 2018; Zhang et al., 2020). Therefore, the downstream mechanism needs to be addressed in future studies as a next step to clarify the molecular target of acupuncture.

In summary, the present study reveals that acupuncture protects short-term memory and spatial and learning memory of BCCAO rats through increasing subcortical white matter perfusion and improving white matter integrity. The attenuation of myelin loss and microglia-associated neuroinflammation in part underlies the beneficial effects of acupuncture on white matter lesions. Intensive studies of downstream neural circuits that mediate the stimulating signals are encouraged to confirm the therapeutic effects of acupuncture in VaD.

## Data Availability Statement

The raw data supporting the conclusions of this article will be made available by the authors, without undue reservation.

## Ethics Statement

The animal study was reviewed and approved by Institutional Animal Care and Use Committee of the Dongfang hospital, Beijing University of Chinese Medicine.

## Author Contributions

S-MM performed imaging processing and pathological staining and drafted the manuscript. LW, N-NY, and X-TS established the animal model and performed the behavioral test. JH and L-LL analyzed behavioral test data and pathological staining. J-KS participated in the ASL imaging and partial DTI imaging processing. C-ZL and J-WY were responsible for designing and supervising the study. All authors critically reviewed and approved the manuscript.

## Conflict of Interest

The authors declare that the research was conducted in the absence of any commercial or financial relationships that could be construed as a potential conflict of interest.

## Footnotes

1 http://dsi-studio.labsolver.org

## References

[B1] Adams D. Cheng F. Jou H. Aung S. Yasui Y. Vohra S. (2011). The safety of pediatric acupuncture: a systematic review. *Pediatrics* 128 E1575–E1587. 10.1542/peds.2011-1091 22106073

[B2] Alber J. Alladi S. Bae H. J. Barton D. A. Beckett L. A. Bell J. M. (2019). White matter hyperintensities in vascular contributions to cognitive impairment and dementia (VCID): knowledge gaps and opportunities. *Alzheimers Dement.* 5 107–117. 10.1016/j.trci.2019.02.001 31011621 PMC6461571

[B3] Alfaro F. J. Gavrieli A. Saade-Lemus P. Lioutas V. A. Upadhyay J. Novak V. (2018). White matter microstructure and cognitive decline in metabolic syndrome: a review of diffusion tensor imaging. *Metabolism* 78 52–68. 10.1016/j.metabol.2017.08.009 28920863 PMC5732847

[B4] Basser P. J. Mattiello J. Lebihan D. (1994). Estimation of the effective self-diffusion tensor from the nmr spin-echo. *J. Magnet. Reson. Ser. B* 103 247–254. 10.1006/jmrb.1994.1037 8019776

[B5] Ben-Ari H. Lifschyz T. Wolf G. Rigbi A. Blumenfeld-Katzir T. Merzel T. K. (2019). White matter lesions, cerebral inflammation and cognitive function in a mouse model of cerebral hypoperfusion. *Brain Res.* 1711 193–201. 10.1016/j.brainres.2019.01.017 30659829

[B6] Bevins R. A. Besheer J. (2006). Object recognition in rats and mice: a one-trial non-matching-to-sample learning task to study ‘recognition memory’. *Nat. Protoc.* 1 1306–1311. 10.1038/nprot.2006.205 17406415

[B7] Calabrese V. Giordano J. Signorile A. Ontario M. L. Castorina S. De Pasquale C. (2016). Major pathogenic mechanisms in vascular dementia: roles of cellular stress response and hormesis in neuroprotection. *J. Neurosci. Res.* 94 1588–1603. 10.1002/jnr.23925 27662637

[B8] Choi B.-R. Kim D.-H. Bin Back D. Kang C. H. Moon W.-J. Han J.-S. (2016). Characterization of white matter injury in a rat model of chronic cerebral hypoperfusion. *Stroke* 47 542–547. 10.1161/strokeaha.115.011679 26670084

[B9] Cui S. Xu M. Huang J. Wang Q. M. Lai X. Nie B. (2018). Cerebral responses to acupuncture at GV24 and Bilateral GB13 in Rat Models of Alzheimer’s Disease. *Behav. Neurol.* 2018:8740284. 10.1155/2018/8740284 29854022 PMC5952587

[B10] Datta G. Colasanti A. Rabiner E. A. Gunn R. N. Malik O. Ciccarelli O. (2017). Neuroinflammation and its relationship to changes in brain volume and white matter lesions in multiple sclerosis. *Brain* 140 2927–2938. 10.1093/brain/awx228 29053775

[B11] Dhond R. P. Yeh C. Park K. Kettner N. Napadow V. (2008). Acupuncture modulates resting state connectivity in default and sensorimotor brain networks. *Pain* 136 407–418. 10.1016/j.pain.2008.01.011 18337009 PMC2440647

[B12] Dichgans M. Leys D. (2017). Vascular cognitive impairment. *Circ. Res.* 120 573–591. 10.1161/circresaha.116.308426 28154105

[B13] Ding N. Jiang J. Xu A. Tang Y. Li Z. (2019). Manual acupuncture regulates behavior and cerebral blood flow in the SAMP8 mouse model of Alzheimer’s Disease. *Front. Neurosci.* 13:37. 10.3389/fnins.2019.00037 30766475 PMC6365452

[B14] Dong Y. F. Kataoka K. Toyama K. Sueta D. Koibuchi N. Yamamoto E. (2011). Attenuation of brain damage and cognitive impairment by direct renin inhibition in mice with chronic cerebral hypoperfusion. *Hypertension* 58 635–642. 10.1161/hypertensionaha.111.173534 21859961

[B15] Du S.-Q. Wang X.-R. Xiao L.-Y. Tu J.-F. Zhu W. He T. (2017). Molecular mechanisms of vascular dementia: what can be learned from animal models of chronic cerebral hypoperfusion? *Mol. Neurobiol.* 54 3670–3682. 10.1007/s12035-016-9915-1 27206432

[B16] Faraci F. M. (2011). Protecting against vascular disease in brain. *Am. J. Physiol. Heart Circ. Physiol.* 300 H1566–H1582. 10.1152/ajpheart.01310.2010 21335467 PMC3094081

[B17] Fu J.-L. Zhang T. Chang C. Zhang Y.-Z. Li W.-B. (2012). The value of diffusion tensor imaging in the differential diagnosis of subcortical ischemic vascular dementia and Alzheimer’s disease in patients with only mild white matter alterations on T2-weighted images. *Acta Radiol.* 53 312–317. 10.1258/ar.2011.110272 22416261

[B18] Giezendanner S. Fisler M. S. Soravia L. M. Andreotti J. Walther S. Wiest R. (2016). Microstructure and cerebral blood flow within white matter of the human brain: a TBSS analysis. *PLoS One* 11:e0150657. 10.1371/journal.pone.0150657 26942763 PMC4778945

[B19] Gorelick P. B. Scuteri A. Black S. E. DeCarli C. Greenberg S. M. Iadecola C. (2011). Vascular contributions to cognitive impairment and dementia a statement for healthcare professionals from the american heart association/american stroke association. *Stroke* 42 2672–2713. 10.1161/STR.0b013e3182299496 21778438 PMC3778669

[B20] Hattori Y. Enmi J.-I. Iguchi S. Saito S. Yamamoto Y. Nagatsuka K. (2016). Substantial reduction of parenchymal cerebral blood flow in mice with bilateral common carotid artery stenosis. *Sci. Rep.* 6:32179. 10.1038/srep32179 27535801 PMC4989493

[B21] Iadecola C. (2013). The pathobiology of vascular dementia. *Neuron* 80 844–866. 10.1016/j.neuron.2013.10.008 24267647 PMC3842016

[B22] Kim Y. J. Kwon H. K. Lee J. M. Kim Y. J. Kim H. J. Jung N. Y. (2015). White matter microstructural changes in pure Alzheimer’s disease and subcortical vascular dementia. *Eur. J. Neurol.* 22 709–716. 10.1111/ene.12645 25603760

[B23] Miyamoto N. Maki T. Pham L. D. Hayakawa K. Seo J. H. Mandeville E. T. (2013). Oxidative stress interferes with white matter renewal after prolonged cerebral hypoperfusion in mice. *Stroke* 44 3516–3521. 10.1161/strokeaha.113.002813 24072001 PMC3985753

[B24] Ogoh S. (2017). Relationship between cognitive function and regulation of cerebral blood flow. *J. Physiol. Sci.* 67 345–351. 10.1007/s12576-017-0525-0 28155036 PMC10717011

[B25] Palesi F. De Rinaldis A. Vitali P. Castellazzi G. Casiraghi L. Germani G. (2018). Specific Patterns of White Matter Alterations Help Distinguishing Alzheimer’s and Vascular Dementia. *Front. Neurosci.* 12:274. 10.3389/fnins.2018.00274 29922120 PMC5996902

[B26] Qin C. Liu Q. Hu Z. W. Zhou L. Q. Shang K. Bosco D. B. (2018). Microglial TLR4-dependent autophagy induces ischemic white matter damage via STAT1/6 pathway. *Theranostics* 8 5434–5451. 10.7150/thno.27882 30555556 PMC6276098

[B27] Qiu W. Q. Claunch J. Kong J. Nixon E. E. Fang J. Li M. (2010). The effects of acupuncture on the brain networks for emotion and cognition: an observation of gender differences. *Brain Res.* 1362 56–67. 10.1016/j.brainres.2010.09.040 20851113 PMC3784241

[B28] Reijmer Y. D. Schultz A. P. Leemans A. O’Sullivan M. J. Gurol M. E. Sperling R. (2015). Decoupling of structural and functional brain connectivity in older adults with white matter hyperintensities. *Neuroimage* 117 222–229. 10.1016/j.neuroimage.2015.05.054 26025290 PMC4511724

[B29] Saggu R. Schumacher T. Gerich F. Rakers C. Tai K. Delekate A. (2016). Astroglial NF-kB contributes to white matter damage and cognitive impairment in a mouse model of vascular dementia. *Acta Neuropathol. Commun.* 4:76. 10.1186/s40478-016-0350-3 27487766 PMC4973061

[B30] Schuff N. Matsumoto S. Kmiecik J. Studholme C. Du A. Ezekiel F. (2009). Cerebral blood flow in ischemic vascular dementia and Alzheimer’s disease, measured by arterial spin-labeling magnetic resonance imaging. *Alzheimers Dement.* 5 454–462. 10.1016/j.jalz.2009.04.1233 19896584 PMC2802181

[B31] Sharma R. Fischer M. T. Bauer J. Felts P. A. Smith K. J. Misu T. (2010). Inflammation induced by innate immunity in the central nervous system leads to primary astrocyte dysfunction followed by demyelination. *Acta Neuropathol.* 120 223–236. 10.1007/s00401-010-0704-z 20532539 PMC2892605

[B32] Shi G.-X. Liu C.-Z. Guan W. Wang Z.-K. Wang L. Xiao C. (2014). Effects of acupuncture on Chinese medicine syndromes of vascular dementia. *Chin. J. Integr. Med.* 20 661–666. 10.1007/s11655-013-1323-4 24155069

[B33] Struys T. Govaerts K. Oosterlinck W. Casteels C. Bronckaers A. Koole M. (2017). In vivo evidence for long-term vascular remodeling resulting from chronic cerebral hypoperfusion in mice. *J. Cereb. Blood Flow Metab.* 37 726–739. 10.1177/0271678x16638349 26994041 PMC5381461

[B34] Torres-Rosas R. Yehia G. Peña G. Mishra P. del Rocio Thompson-Bonilla M. Moreno-Eutimio M. A. (2014). Dopamine mediates vagal modulation of the immune system by electroacupuncture. *Nat. Med.* 20 291–295. 10.1038/nm.3479 24562381 PMC3949155

[B35] Uchida S. Kagitani F. (2015). Effect of acupuncture-like stimulation on cortical cerebral blood flow in aged rats. *J. Physiol. Sci.* 65 67–75. 10.1007/s12576-014-0340-9 25300864 PMC10717680

[B36] Wang S. Liu K. Wang Y. Wang S. He X. Cui X. (2017). A proposed neurologic pathway for scalp acupuncture: trigeminal nerve-meninges-cerebrospinal fluid-contacting neurons-brain. *Med. Acupunct.* 29 322–326. 10.1089/acu.2017.1231 29067143 PMC5653342

[B37] Wang X. Lin F. Gao Y. Lei H. (2015). Bilateral common carotid artery occlusion induced brain lesions in rats: a longitudinal diffusion tensor imaging study. *Magn. Reson. Imaging* 33 551–558. 10.1016/j.mri.2015.02.010 25708261

[B38] Wang X. R. Shi G. X. Yang J. W. Yan C. Q. Lin L. T. Du S. Q. (2015). Acupuncture ameliorates cognitive impairment and hippocampus neuronal loss in experimental vascular dementia through Nrf2-mediated antioxidant response. *Free Radic. Biol. Med.* 89 1077–1084. 10.1016/j.freeradbiomed.2015.10.426 26546103

[B39] Wang Z. Williams V. J. Stephens K. A. Kim C. M. Bai L. Zhang M. (2020). The effect of white matter signal abnormalities on default mode network connectivity in mild cognitive impairment. *Hum. Brain Mapp.* 41 1237–1248. 10.1002/hbm.24871 31742814 PMC7267894

[B40] Watson C. Paxinos G. (1996). *The Rat Brain in Stereotaxic Coordinates*, 2nd Edn Cambridge, MA: Academic Press.

[B41] Wen T. Zhang X. Liang S. Li Z. Xing X. Liu W. (2018). Electroacupuncture ameliorates cognitive impairment and spontaneous low-frequency brain activity in rats with ischemic stroke. *J. Stroke Cerebrovasc. Dis.* 27 2596–2605. 10.1016/j.jstrokecerebrovasdis.2018.05.021 30220306

[B42] Xiao L. Y. Wang X. R. Yang J. W. Ye Y. Zhu W. Cao Y. (2018). Acupuncture prevents the impairment of hippocampal LTP Through beta1-AR in vascular dementia rats. *Mol. Neurobiol.* 55 7677–7690. 10.1007/s12035-018-0943-x 29435917

[B43] Ye Y. Li H. Yang J.-W. Wang X.-R. Shi G.-X. Yan C.-Q. (2017). Acupuncture attenuated vascular dementia-induced hippocampal long-term potentiation impairments via activation of D1/D5 receptors. *Stroke* 48 1044–1051. 10.1161/strokeaha.116.014696 28289242

[B44] Yu J. C. Zhang X. Z. Liu C. Z. Meng Y. C. Han J. X. (2006). Effect of acupuncture treatment on vascular dementia. *Neurol. Res.* 28 97–103. 10.1179/016164106x91951 16464371

[B45] Zhang L.-Y. Pan J. Mamtilahun M. Zhu Y. Wang L. Venkatesh A. (2020). Microglia exacerbate white matter injury via complement C3/C3aR pathway after hypoperfusion. *Theranostics* 10 74–90. 10.7150/thno.35841 31903107 PMC6929610

[B46] Zhang L. Y. Pan J. Mamtilahun M. Zhu Y. Wang L. Venkatesh A. (2020). Microglia exacerbate white matter injury via complement C3/C3aR pathway after hypoperfusion. *Theranostics* 10 74–90. 10.7150/thno.35841 31903107 PMC6929610

[B47] Zhang X. Wu B. Nie K. Jia Y. Yu J. (2014). Effects of acupuncture on declined cerebral blood flow, impaired mitochondrial respiratory function and oxidative stress in multi-infarct dementia rats. *Neurochem. Int.* 65 23–29. 10.1016/j.neuint.2013.12.004 24361538

