# Supplementary material for: Acupuncture Improves White Matter Perfusion and Integrity in Rat Model of Vascular Dementia: An MRI-Based Imaging Study
Source: Front Aging Neurosci. 2020 Nov 23;12:582904. doi: 10.3389/fnagi.2020.582904 (PMC7719770; doi:10.3389/fnagi.2020.582904)
Supplement: Supplementary file 1 [file Table_1.DOCX]

Supplemental information

**Acupuncture improves white matter perfusion and integrity in rats with vascular dementia: an MRI-based imaging study**

Si-Ming Ma^1^, Lu Wang^2^, Na-Na, Yang^2^, Xin-Tong Su^2^, Jin Huang^1^, Lu-Lu Lin^2^, Jia-Kai Shao^1^, Jing-Wen Yang ^2*^, Cun-Zhi Liu^2^

**Table S1. FA, RD, AD and MD values of white matter regions in rats**

|  |  | 1 | 2 | 3 | 4 | 5 | 6 | 7 | 8 |
| --- | --- | --- | --- | --- | --- | --- | --- | --- | --- |
| FA in CC | **Sham** | 0.73198 | 0.66560 | 0.70823 | 0.70749 | 0.67120 | 0.75113 | 0.71963 | 0.74947 |
|  | **BCCAO** | 0.55156 | 0.54861 | 0.54859 | 0.49157 | 0.50548 | 0.55378 | 0.55875 | 0.51539 |
|  | **BCCAO+Acu** | 0.64700 | 0.67069 | 0.66630 | 0.64953 | 0.74442 | 0.74957 | 0.71600 | 0.72605 |
|  | **BCCAO+Nonacu** | 0.61574 | 0.56609 | 0.42056 | 0.67941 | 0.54324 | 0.49751 | 0.58142 | 0.59354 |
| RD in CC | **Sham** | 0.36829 | 0.42172 | 0.40138 | 0.38675 | 0.48615 | 0.32586 | 0.39969 | 0.37320 |
|  | **BCCAO** | 0.65556 | 0.53270 | 0.54465 | 0.72861 | 0.53606 | 0.55222 | 0.57320 | 0.59076 |
|  | **BCCAO+Acu** | 0.47837 | 0.42530 | 0.51531 | 0.51795 | 0.33953 | 0.33153 | 0.39745 | 0.39022 |
|  | **BCCAO+Nonacu** | 0.51586 | 0.51237 | 0.72053 | 0.46211 | 0.65344 | 0.61430 | 0.49810 | 0.55280 |
| AD in CC | **Sham** | 1.72776 | 1.55428 | 1.78713 | 1.60002 | 1.82715 | 1.52052 | 1.73329 | 1.80356 |
|  | **BCCAO** | 1.71644 | 1.44731 | 1.47932 | 1.62223 | 1.27056 | 1.54223 | 1.49973 | 1.45698 |
|  | **BCCAO+Acu** | 1.60006 | 1.54881 | 1.80520 | 1.76360 | 1.53349 | 1.53427 | 1.67911 | 1.72961 |
|  | **BCCAO+Nonacu** | 1.65736 | 1.45624 | 1.35925 | 1.84732 | 1.68577 | 1.43024 | 1.45394 | 1.64570 |
| MD in CC | **Sham** | 0.82144 | 0.79924 | 0.86330 | 0.79118 | 0.93315 | 0.72408 | 0.84422 | 0.84999 |
|  | **BCCAO** | 1.00919 | 0.83757 | 0.85621 | 1.02648 | 0.78090 | 0.88222 | 0.88204 | 0.87950 |
|  | **BCCAO+Acu** | 0.85226 | 0.79980 | 0.94527 | 0.93317 | 0.73751 | 0.73244 | 0.82467 | 0.83668 |
|  | **BCCAO+Nonacu** | 0.89636 | 0.82699 | 0.93343 | 0.92384 | 0.99755 | 0.88628 | 0.81671 | 0.91710 |

|  |  | 1 | 2 | 3 | 4 | 5 | 6 | 7 | 8 |
| --- | --- | --- | --- | --- | --- | --- | --- | --- | --- |
| FA in EC | **Sham** | 0.45590 | 0.44870 | 0.39890 | 0.46009 | 0.43200 | 0.43856 | 0.40862 | 0.45590 |
|  | **BCCAO** | 0.35252 | 0.33651 | 0.36548 | 0.33097 | 0.30899 | 0.32728 | 0.30240 | 0.35252 |
|  | **BCCAO+Acu** | 0.40681 | 0.39463 | 0.38083 | 0.39483 | 0.48650 | 0.50698 | 0.44047 | 0.40681 |
|  | **BCCAO+Nonacu** | 0.34920 | 0.38216 | 0.23800 | 0.38113 | 0.39751 | 0.32495 | 0.32951 | 0.34920 |
| RD in EC | **Sham** | 0.51455 | 0.55099 | 0.56918 | 0.48695 | 0.58653 | 0.35039 | 0.51399 | 0.51455 |
|  | **BCCAO** | 0.61012 | 0.61109 | 0.64140 | 0.61354 | 0.60109 | 0.66090 | 0.60282 | 0.61012 |
|  | **BCCAO+Acu** | 0.57997 | 0.60135 | 0.62193 | 0.56661 | 0.34293 | 0.34649 | 0.54844 | 0.57997 |
|  | **BCCAO+Nonacu** | 0.63208 | 0.58499 | 0.57975 | 0.62217 | 0.62309 | 0.57587 | 0.60062 | 0.63208 |
| AD in EC | **Sham** | 1.07843 | 1.16091 | 1.06927 | 1.05497 | 1.19755 | 0.69216 | 0.99889 | 1.07843 |
|  | **BCCAO** | 1.07398 | 1.03270 | 1.15278 | 1.04617 | 0.93513 | 1.10153 | 0.93221 | 1.07398 |
|  | **BCCAO+Acu** | 1.07398 | 1.03270 | 1.15278 | 1.04617 | 0.93513 | 1.10153 | 0.93221 | 1.07398 |
|  | **BCCAO+Nonacu** | 1.09055 | 1.07690 | 0.84411 | 1.16741 | 1.19236 | 0.96225 | 0.99792 | 1.09055 |
| MD in EC | **Sham** | 0.70251 | 0.75430 | 0.73588 | 0.67629 | 0.79021 | 0.46432 | 0.67563 | 0.70251 |
|  | **BCCAO** | 0.76474 | 0.75163 | 0.81186 | 0.75775 | 0.71244 | 0.80777 | 0.71261 | 0.76474 |
|  | **BCCAO+Acu** | 0.74804 | 0.76852 | 0.77982 | 0.73074 | 0.47345 | 0.48916 | 0.74208 | 0.74804 |
|  | **BCCAO+Nonacu** | 0.78490 | 0.74896 | 0.66787 | 0.80392 | 0.81285 | 0.70466 | 0.73305 | 0.78490 |

|  |  | 1 | 2 | 3 | 4 | 5 | 6 | 7 | 8 |
| --- | --- | --- | --- | --- | --- | --- | --- | --- | --- |
| FA in IC | **Sham** | 0.63256 | 0.61094 | 0.57654 | 0.61082 | 0.65211 | 0.65565 | 0.64495 | 0.65319 |
|  | **BCCAO** | 0.58110 | 0.56965 | 0.59714 | 0.56741 | 0.57046 | 0.57717 | 0.57175 | 0.58784 |
|  | **BCCAO+Acu** | 0.57645 | 0.69230 | 0.65938 | 0.61000 | 0.63446 | 0.67361 | 0.67782 | 0.66387 |
|  | **BCCAO+Nonacu** | 0.61177 | 0.56122 | 0.50361 | 0.56975 | 0.54123 | 0.55249 | 0.61683 | 0.56039 |
| RD in IC | **Sham** | 0.41544 | 0.44348 | 0.44706 | 0.45075 | 0.45347 | 0.37423 | 0.41182 | 0.41515 |
|  | **BCCAO** | 0.46471 | 0.48731 | 0.45046 | 0.47713 | 0.47233 | 0.47784 | 0.45062 | 0.44364 |
|  | **BCCAO+Acu** | 0.46590 | 0.49534 | 0.51190 | 0.48575 | 0.50357 | 0.48780 | 0.50752 | 0.54375 |
|  | **BCCAO+Nonacu** | 0.43899 | 0.48357 | 0.48033 | 0.48246 | 0.48374 | 0.47724 | 0.41207 | 0.45885 |
| AD in IC | **Sham** | 1.35154 | 1.37308 | 1.25113 | 1.38784 | 1.55844 | 1.27476 | 1.37072 | 1.43753 |
|  | **BCCAO** | 1.34301 | 1.34398 | 1.32700 | 1.30277 | 1.29712 | 1.31424 | 1.23519 | 1.18713 |
|  | **BCCAO+Acu** | 1.27991 | 1.47727 | 1.31738 | 1.23777 | 1.30352 | 1.31568 | 1.39614 | 1.37496 |
|  | **BCCAO+Nonacu** | 1.35597 | 1.30758 | 1.13711 | 1.33592 | 1.24185 | 1.25489 | 1.27017 | 1.21402 |
| MD in IC | **Sham** | 0.72748 | 0.75335 | 0.71509 | 0.76311 | 0.82180 | 0.67440 | 0.73145 | 0.75594 |
|  | **BCCAO** | 0.75747 | 0.77287 | 0.74264 | 0.75234 | 0.74726 | 0.75664 | 0.71215 | 0.69147 |
|  | **BCCAO+Acu** | 0.73249 | 0.74529 | 0.69373 | 0.68586 | 0.70285 | 0.68287 | 0.71325 | 0.71940 |
|  | **BCCAO+Nonacu** | 0.74465 | 0.75824 | 0.69925 | 0.76695 | 0.73644 | 0.73646 | 0.69810 | 0.71057 |

|  |  | 1 | 2 | 3 | 4 | 5 | 6 | 7 | 8 |
| --- | --- | --- | --- | --- | --- | --- | --- | --- | --- |
| FA in ON | **Sham** | 0.38790 | 0.40780 | 0.42154 | 0.36985 | 0.36131 | 0.44274 | 0.45969 | 0.34805 |
|  | **BCCAO** | 0.25362 | 0.26913 | 0.28961 | 0.30179 | 0.21715 | 0.26079 | 0.26745 | 0.29748 |
|  | **BCCAO+Acu** | 0.28565 | 0.36990 | 0.35583 | 0.31071 | 0.26442 | 0.46723 | 0.44639 | 0.40903 |
|  | **BCCAO+Nonacu** | 0.22677 | 0.27394 | 0.29973 | 0.28055 | 0.25122 | 0.29530 | 0.30951 | 0.27996 |
| RD in ON | **Sham** | 1.35692 | 1.03886 | 1.51698 | 1.35704 | 1.46219 | 0.97204 | 1.04145 | 0.90154 |
|  | **BCCAO** | 1.71058 | 1.63320 | 1.50510 | 1.80595 | 1.74375 | 1.61863 | 1.75622 | 1.31168 |
|  | **BCCAO+Acu** | 1.43862 | 1.51471 | 1.51485 | 1.33312 | 1.23813 | 1.10141 | 1.24061 | 1.70547 |
|  | **BCCAO+Nonacu** | 1.73549 | 1.30757 | 0.89759 | 1.15617 | 1.39948 | 1.01310 | 1.47101 | 1.52467 |
| AD in ON | **Sham** | 2.65080 | 2.93368 | 3.03363 | 2.34233 | 2.23494 | 2.15933 | 2.63799 | 2.83552 |
|  | **BCCAO** | 2.55507 | 2.41199 | 2.34846 | 2.95922 | 2.46498 | 2.40513 | 2.59504 | 1.95976 |
|  | **BCCAO+Acu** | 2.09154 | 1.75977 | 2.47654 | 2.07441 | 2.41759 | 1.99669 | 2.13011 | 1.76588 |
|  | **BCCAO+Nonacu** | 2.43791 | 1.94411 | 2.44624 | 2.37503 | 1.96197 | 1.61491 | 2.34706 | 2.29802 |
| MD in ON | **Sham** | 1.84268 | 1.98770 | 1.02111 | 1.66953 | 1.57040 | 1.45405 | 1.70640 | 1.08215 |
|  | **BCCAO** | 1.99208 | 1.89280 | 1.78622 | 2.19037 | 1.98416 | 1.88080 | 2.03583 | 1.52771 |
|  | **BCCAO+Acu** | 1.60179 | 1.27916 | 1.86683 | 1.59616 | 1.90065 | 1.31359 | 1.40434 | 1.89650 |
|  | **BCCAO+Nonacu** | 1.96963 | 1.51975 | 1.08047 | 1.37503 | 1.58698 | 1.21371 | 1.76303 | 1.78246 |

|  |  | 1 | 2 | 3 | 4 | 5 | 6 | 7 | 8 |
| --- | --- | --- | --- | --- | --- | --- | --- | --- | --- |
| FA in OT | **Sham** | 0.57674 | 0.54886 | 0.52058 | 0.56580 | 0.53064 | 0.54162 | 0.56920 | 0.53354 |
|  | **BCCAO** | 0.41882 | 0.38109 | 0.44931 | 0.41047 | 0.35974 | 0.41387 | 0.44303 | 0.34776 |
|  | **BCCAO+Acu** | 0.44519 | 0.43381 | 0.44898 | 0.36305 | 0.53747 | 0.53077 | 0.47970 | 0.46309 |
|  | **BCCAO+Nonacu** | 0.34015 | 0.38353 | 0.44120 | 0.41854 | 0.36184 | 0.36262 | 0.45811 | 0.48391 |
| RD in OT | **Sham** | 0.46590 | 0.49534 | 0.51190 | 0.58575 | 0.60357 | 0.48780 | 0.50752 | 0.54375 |
|  | **BCCAO** | 0.61987 | 0.71652 | 0.64199 | 0.69881 | 0.83881 | 0.88244 | 0.65327 | 0.83725 |
|  | **BCCAO+Acu** | 0.46590 | 0.49534 | 0.51190 | 0.58575 | 0.60357 | 0.48780 | 0.50752 | 0.54375 |
|  | **BCCAO+Nonacu** | 0.79250 | 0.60920 | 0.54538 | 0.64060 | 0.66545 | 0.63567 | 0.77371 | 0.54528 |
| AD in OT | **Sham** | 1.27454 | 1.25627 | 1.23594 | 1.55529 | 1.47654 | 1.21817 | 1.34012 | 1.35444 |
|  | **BCCAO** | 1.22767 | 1.28329 | 1.33104 | 1.31808 | 1.44344 | 1.72156 | 1.34953 | 1.43148 |
|  | **BCCAO+Acu** | 1.13253 | 1.32008 | 1.20313 | 1.07561 | 1.18239 | 1.28212 | 1.58175 | 1.33598 |
|  | **BCCAO+Nonacu** | 1.32341 | 1.12050 | 1.09740 | 1.23943 | 1.11939 | 1.11186 | 1.64081 | 1.23433 |
| MD in OT | **Sham** | 0.73545 | 0.74898 | 0.52058 | 0.90893 | 0.89456 | 0.73126 | 0.78505 | 0.81398 |
|  | **BCCAO** | 0.82247 | 0.90544 | 0.87168 | 0.90523 | 1.04035 | 1.16215 | 0.88536 | 1.03532 |
|  | **BCCAO+Acu** | 0.75292 | 0.87769 | 0.79248 | 0.76140 | 0.71020 | 0.78145 | 1.02204 | 0.85824 |
|  | **BCCAO+Nonacu** | 0.96947 | 0.77963 | 0.72939 | 0.84021 | 0.81676 | 0.79440 | 1.06274 | 0.77497 |

FA: fractional anisotropy, RD: radial diffusivity, AD: axial diffusivity, MD: mean diffusivity, CC: corpus callosum, EC: external capsule, IC: internal capsule, ON: optic nerve, OT: optic tract, n=8 per group. SHAM: sham-operated group, BCCAO: BCCAO-operated group, BCCAO+ACU: BCCAO-operated + acupuncture at GV20 and ST36 group, BCCAO+NON-ACU: BCCAO-operated + acupuncture at non-acupoints group.

|  | CBF | FA | RD | Fiber density |
| --- | --- | --- | --- | --- |
| SHAM 1 | 28.9 | 0.75113 | 0.72861 | 1.57042 |
| SHAM 2 | 20.8 | 0.74957 | 0.72053 | 1.37333 |
| SHAM 3 | 22.3 | 0.74947 | 0.65556 | 1.31250 |
| SHAM 4 | 25.2 | 0.74442 | 0.65344 | 1.19271 |
| SHAM 5 | 17.9 | 0.73198 | 0.61430 | 1.15419 |
| SHAM 6 | 16.3 | 0.72605 | 0.59076 | 1.14876 |
| SHAM 7 | 19.9 | 0.71963 | 0.57320 | 1.13426 |
| SHAM 8 | 14.4 | 0.71600 | 0.55280 | 1.12778 |
| BCCAO 1 | 19.3 | 0.70823 | 0.55222 | 1.11310 |
| BCCAO 2 | 12 | 0.70749 | 0.54465 | 1.06218 |
| BCCAO 3 | 18.8 | 0.67941 | 0.53606 | 1.06044 |
| BCCAO 4 | 17 | 0.67120 | 0.53270 | 1.04167 |
| BCCAO 5 | 16.9 | 0.67069 | 0.51795 | 1.01058 |
| BCCAO 6 | 7.15 | 0.66630 | 0.51586 | 1.01026 |
| BCCAO 7 | 6.79 | 0.66560 | 0.51531 | 1.00870 |
| BCCAO 8 | 7.33 | 0.64953 | 0.51237 | 0.97980 |
| BCCAO+ACU 1 | 22.3 | 0.64700 | 0.49810 | 0.97980 |
| BCCAO+ACU 2 | 27.7 | 0.61574 | 0.48615 | 0.96970 |
| BCCAO+ACU 3 | 23.1 | 0.59354 | 0.47837 | 0.94526 |
| BCCAO+ACU 4 | 17.9 | 0.58142 | 0.46211 | 0.93802 |
| BCCAO+ACU 5 | 18 | 0.56609 | 0.42530 | 0.93103 |
| BCCAO+ACU 6 | 20.4 | 0.55875 | 0.42172 | 0.92381 |
| BCCAO+ACU 7 | 16.37 | 0.55378 | 0.40138 | 0.91936 |
| BCCAO+ACU 8 | 15.9 | 0.55156 | 0.39969 | 0.86408 |
| BCCAO+NON-ACU 1 | 17.8 | 0.54861 | 0.39745 | 0.85841 |
| BCCAO+NON-ACU 2 | 19.9 | 0.54859 | 0.39022 | 0.83920 |
| BCCAO+NON-ACU 3 | 14.2 | 0.54324 | 0.38675 | 0.83598 |
| BCCAO+NON-ACU 4 | 15 | 0.51539 | 0.37320 | 0.82573 |
| BCCAO+NON-ACU 5 | 13.1 | 0.50548 | 0.36829 | 0.82212 |
| BCCAO+NON-ACU 6 | 9.6 | 0.49751 | 0.33953 | 0.79918 |
| BCCAO+NON-ACU 7 | 11.2 | 0.49157 | 0.33153 | 0.77619 |
| BCCAO+NON-ACU 8 | 6.79 | 0.42056 | 0.32586 | 0.55122 |

**Table S2.** **The values of CBF, FA, RD and fiber density in corpus callosum of each rat**

FA: fractional anisotropy, RD: radial diffusivity, n=8 per group. SHAM: sham-operated group, BCCAO: BCCAO-operated group, BCCAO+ACU: BCCAO-operated + acupuncture at GV20 and ST36 group, BCCAO+NON-ACU: BCCAO-operated + acupuncture at non-acupoints group.
